# Time Delay for Aerial Ammonia Concentration Measurements in Livestock Buildings

**DOI:** 10.3390/s100504634

**Published:** 2010-05-04

**Authors:** Hans Benny Rom, Guo-Qiang Zhang

**Affiliations:** Department of Biosystems Engineering, University of Aarhus, Faculty of Agricultural Sciences, Blichers Alle 20, DK-8830 Tjele, Denmark; E-Mail: GuoQiang.Zhang@agrsci.dk

**Keywords:** photo acoustic spectroscopy, ammonia measurement, time delay

## Abstract

Correct measurements of ammonia concentration in air still present considerable challenges. The high water solubility and polarity can cause it to adsorb on surfaces in the entire sampling system, including sampling lines, filters, valves, pumps and instruments, causing substantial measuring errors and time delays. To estimate time delay characteristics of a Photo Acoustic Multi Gas Monitor 1312 and a Multi Point Sampler continuous measurement of aerial ammonia concentrations at different levels was performed. In order to obtain reproducible data, a wind tunnel was used to generate selected concentrations inside and a background concentration representing the air inlet of the tunnel. Four different concentration levels (0.8 ppm, 6.2 ppm, 9.7 ppm and 13.7 ppm) were used in the experiments, with an additional outdoor concentration level as background. The results indicated a substantial time delay when switching between the measuring positions with high and low concentration and *vice versa*. These properties may course serious errors for estimation of e.g. gas emissions whenever more than one measuring channel is applied. To reduce the measurement errors, some suggestions regarding design of the measurement setup and measuring strategies were presented.

## 1. Introduction

Correct measurement of ammonia concentrations in air still present considerable challenges, due to the properties of ammonia and state of the art of available technologies. The high water solubility and polarity can cause it to adsorb on surfaces throughout the entire sampling system, including sampling lines, filters, valves, pumps and instruments, with subsequent serious consequences such as time delays, measurement and calculation errors, *etc*.

Several measurement systems and principles are described in the literature for detecting aerial ammonia concentrations in the agricultural systems [1]. The instruments have different detection principles and the procedures for doing reliable measurements differ a lot. According to Phillips *et al.* [2] the techniques fall into three categories: detection tubes with a chemical gas specific absorption granulate for instantaneous measurements, accumulative ones that rely on capturing ammonia over time and finally continuous ones equipped with rapidly responding sensors to follow changes in concentration. Detection tubes are relative cheap in purchase but may not be recommendable for measuring concentrations below 2.5 ppm NH_3_ [2]. The capturing detection techniques such as flux denuders, passive flux samplers and adsorption bottles are primarily used for detection of very low concentrations such as NH_3_ dry deposition or ambient air concentration levels. The capturing detectors are relatively cheap to purchase, but have high labour costs. Continuous methods like electrochemical cells, chemiluminescence (NO_x_-analyser), fluorescence, Photo Acoustic Spectroscopy (PAS) and long path optical methods are used for detection of concentration variations such as those found in the measurement of air quality inside livestock buildings and gas emissions from the buildings. The purchase price is high and running costs differ a lot due to different calibration intervals and general maintenance needs. Ni and Heber [1] and Phillips *et al*. [3] have presented details about the agricultural applications and properties of the various systems.

During the last decade an increasing number of papers on NH_3_ emissions have conducted their investigations using PAS instruments such as the Multi-Gas-Monitors and Multipoint Samplers from (Innova) LumaSense Technologies A/S (Denmark). Most of the articles pay little or no attention to data quality parameters such as calibration or quality assessment of the measuring setups used [1,4]. Experiences from use of PAS instruments for measurements of ammonia concentration in air during 15 years show that the instruments need time to indicate correct levels when the ammonia concentration varies from one position to another [5,6].

A number of studies have already focused on ammonia adsorption on various types of tubing materials used in pollutant stream conveyance [7–9]. However, knowledge concerning optimal measurement procedures for a multi-positional data sampling application is missing. Therefore, this paper will focus on the numbers of measurement events or sampling duration and the corresponding accuracy for each position with different ammonia concentration levels to improve the measurement procedures.

## 2. Materials and Methods

### 2.1. Photo Acoustic Spectroscopy Detection Method

**Figure 1.**
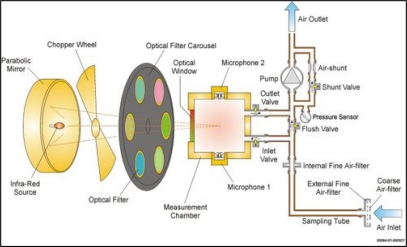
Photo Acoustic Spectroscopy detection principle [10].

A number of optical filters for specific detection of a large number of different gases can be applied to the Multi-Gas-Monitor [10]. Figure 1 illustrates the measurement principle, which begins with an air sample being drawn into the measurement chamber and the chamber being then sealed by valves. Radiation from an IR-source passes through a chopper and an optical filter into the chamber. The radiation energy is absorbed by the specific gas proportionally to the concentration and subsequently converted to heat, which generates pressure waves. Microphones detect these pressure waves and the signal is post processed and the gas concentration can be estimated. [10]

### 2.2. Experimental Setup

A 1312 Multi-Gas-Monitor 1312 and a 1303 [11] or 1309 [12] Multi Point Sampler were used in the studies. The technical data of the optical filters used for gas detection are listed in Table 1.

Osada *et al.* concluded that the Multi Gas Monitor overestimates the CO_2_, N_2_O and CH_4_ levels when measurements were carried out in the lower end of the calibration range [13]. In order to improve the accuracy for monitoring both high or low concentrations the optical CO_2_, N_2_O and CH_4_ filters were calibrated in two different levels and the calibration data were loaded in two different banks in the monitor. As this was not the case for NH_3_ and Freon measurements, the double calibration was only done for the CO_2_, N_2_O and CH_4_ filters.

The Monitor was configured with a chamber flush time of 60 s and Sample Integration Time (SIT) of 5 s for all gases, except ammonia which was set at 60 s. Nevertheless certified calibration is not at guarantee for correct measurements, because the whole measurement chain such as sampling tubes, dust filters transportation length and instrument properties regarding individual gases have an influence on the reliability.

**Figure 2.**
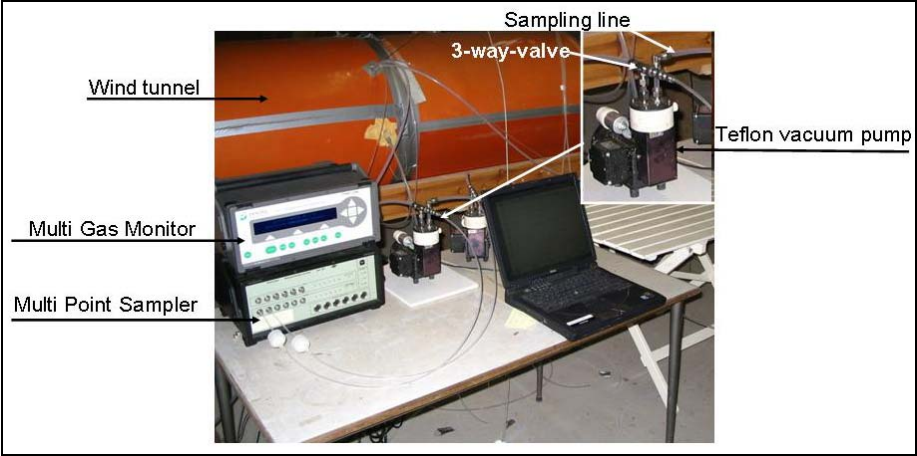
Instrumentation setup.

Figure 2 shows the experimental setup. In order to simulate data collection in practice and to obtain reproducible data a wind tunnel using atmospheric air mixed with clean ammonia was used. The concentration inside the wind tunnel simulates the high concentration position and the outdoor position was used as in the case for data collection in practice. The concentration in the wind tunnel was kept constant during each session of the study. Four sessions were done with ammonia concentration levels of 0.8 ppm, 6.2 ppm, 9.7 ppm and 13.7 ppm. The background level was 0.2–0.3 ppm. The Multi Point Sampler was configured to repeat the measuring event 15 times in the low concentration position before switching to 0.8, 6.2, 9.7 or 13.7 ppm respectively. The cycles of high and low concentration measurements were repeated 14–18 times for concentration ranges up to 9.7 ppm and 4–5 times for 14 ppm. NH_3_ is often measured simultaneously with CO_2_, N_2_O and CH_4_, which is the reason for including them in this study. Experience shows that among these gases NH_3_ is the only one displaying a substantial time delay, which is the reason for studying this specific gas.

The concentrations of 0.8 ppm and 6.2 ppm in the tunnel were validated by means of three parallel sets of absorption bottles with a strong acid solution. The absorbent was a 0.002 M phosphoric acid (H_3_PO_4_) solution and the air flow through the bottles was 2 L/min.

### 2.3. Air Conveyance from the Tunnel to the Instrument

A tetraflouroethylene-hexaflouropropylene (FEP) tube of Ø 3/4.8 mm and Ø 6/8 mm (inside/outside diameter) was used for air sampling due to its very low adsorption of ammonia. In order to minimise the time delay caused by the length of the sampling line a type ASF 7015 TF Teflon vacuum pump with bypass (capacity 6–8 L/min) was used to continuously draw the sampling air close to the Multi Point-Sampler-inlet. A three-way connection was applied between each pump and the Multipoint Sampler to ensure the excessive sampled air could exit and thus avoid over-loading the internal pump in the Gas Monitor.

## 3. Results and Discussion

### 3.1. Results

**Figure 3.**
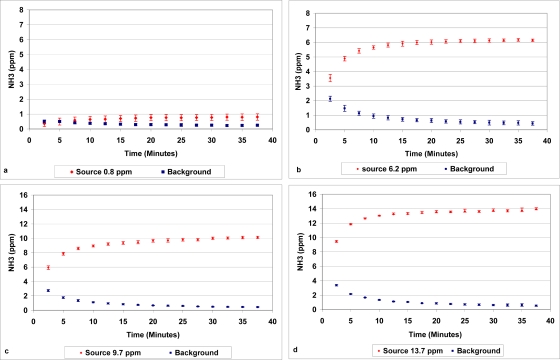
Time delay when switching between high and low concentration positions **(a)** = 0.8 ppm; **(b)** = 6.2 ppm; **(c)** = 9.7 ppm and **(d)** = 13.7 ppm.

All data were obtained for a minimum of two hours after changing the gas concentration level in the tunnel, allowing the gas concentration in the tunnel to stabilize. The overall results are presented in Figure 3. The data shows the transient approach or time delay when switching between high and low concentration levels and *vice versa*. According to the analysis of the adsorption in the bottles the average concentration was 0.8 ppm while the 1312 reading was 0.78 ppm after 37.5 min (15 repetitions) in the same position [Figure 3(a)]. According to the 1312 measurements the background was 0.25 ppm after 37.5 min. Due to the very low concentration difference between tunnel and background the calculated error was rather small.

By increasing the gas concentration of the air in the tunnel to 6.2 ppm the error increased substantially. The average detection concentration for the 1312 was 6.2 ppm after 37.5 min. showing a substantial error during the first 12.5 to 25 min. [Figure 3(b)]. The similar results were found for the concentrations of 10 ppm [Figure 3(c)] and 14 ppm [Figure 3(d)].

### 3.2. Discussion

The results present substantial error due to rapid change between high and low concentration levels. In order to explain the differences between the measured concentrations and the real concentrations a correction factor was derived. The correction factor to be applied when changing concentration from low to high level was derived according to the following equation:

$$C_{f}=1+\frac{V_{t}-V_{m}}{V_{t}}$$

where C_f_ = Correction factor; V_t_ = Target concentration; V_m_ = Measured concentration.

**Figure 4.**
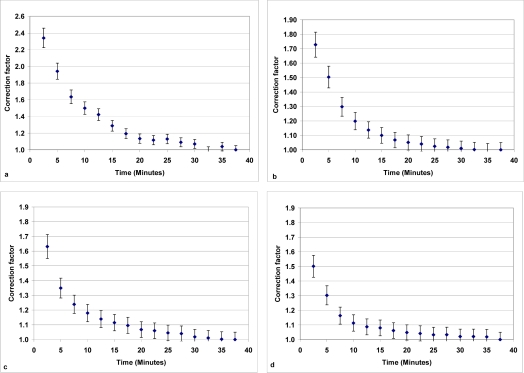
Correction factors with respect to time period in each location **(a)** = 0.8 ppm; **(b)** = 6.2 ppm; **(c)** = 9.7 ppm and **(d)** = 13.7 ppm.

Figure 4 shows the correction factors derived in this study to be used in order to generate a reliable dataset when changing low to high concentrations. In general measuring periods of 12.5 to 25 minutes are needed in order to obtain reliable data. For example, when switching between 0.3 to 6.2 ppm the correction factors increased from 1.02 to 1.14 when the measuring period was reduced from 25 to 12.5 minutes.

There are a number of reasons that could cause the delay. First Ni and Heber [1] and Phillips *et al.* [3] have mentioned that loss of ammonia by adsorption on the inside surfaces of the gas sampling lines and vacuum pumps and by adsorption in condensed water is possible, but these risks were minimized by use of FEP-Teflon and by preventing water condensation by trace heating.

Shah *et al.* [8] have studied a number of tube materials. In that study it was demonstrated that only 0.3% of a total flow of 10 ppm NH_3_ was adsorbed into the FEP-surface. Secondly, if the same tubes were used for sequential sampling of low and high ammonia concentrations, desorption of ammonia from the tubing may result in overestimation of concentration in the low concentration position, at least during the transient period. The opposite is likely to be true when sampling from the high concentration position. In this study separate tubes and vacuum pumps were used for both sampling positions except for the inside surfaces and valves in the Multipoint Sampler and Multigas Monitor and the tube connecting the two units. The total tube length between the Multipoint Sampler and the Multigas Monitor was approximately 0.3 m. In the setup of sampling procedures for the 1312 Multi Gas Monitor it is possible to adjust the flush time for the measuring chamber and for the internal tubes respectively. In this study the flush time was 60 s and 3 s. for the chamber and the tubes. In order to minimise the deviation of the measurements the Sample Integration Time (SIT) can be adjusted from 5 to 60 s for each individual gas. In this case the SIT for the ammonia filter was set to 60 s due to the high water solubility and polarity. The disadvantage of using a longer SIT is that the time required to complete each measurement will increase. On the other hand, shortening the SIT results in an increased deviation, but more measurements can be done. In addition, the design of the measuring chamber could also have some effect on the delay. According to the information provided by the manufacturer the chamber is cylindrical, so it may have some locations that are not properly flushed, consequently causing a memory effect from one event to the next, although this does not seem to be the main reason behind the observed lag, due to the fact that it is mainly NH_3_ which has the time delay problem and other instruments seem to have almost similar responses to NH_3_ such as the NO_x_ analyser with an upstream thermal ammonia converter that has a time delay of up to 4 minutes for the 5.4 to 1.1 ppm range [3]. Unpublished studies carried out in the Climate Laboratory at Institute of Biosystems Engineering, Aarhus University (Denmark), indicated that a NO_x_-analyser with an internal NH_3_-NO_x_ converter had a time delay in the same range as for the Multigas Monitor.

The results indicate that the chosen setup required approximately 37–38 minutes (15 repeating measuring events) in each position, depending of the number of gases to be measured and the setup of the monitor. The time delay is mainly due to adherence effects on the surfaces in the instruments and in the tube between the instruments. It is assumed that the main reason is the surfaces and materials inside the instruments, which are used for sequential sampling of low and high ammonia concentrations. To reduce this effect it could be desirable to improve the instrument setup with an option for selection of longer flush periods for the internal tubes, similar to the functions for Chamber flush and SIT.

Measuring ammonia concentration in air seems to be a question of compromises. When measuring only NH_3_ at various concentration levels, a NO_x_-analyser with an upstream NH_3_-NO_x_-converter could be recommended, but if the aim is to estimate more gases simultaneously then a Multigas Monitor could be an option taking the mentioned disadvantages into account. If the aim is to estimate an average daily level, then passive adsorption methods should be seriously considered. When doing measurements of ammonia concentrations with substantial variation between various measuring positions, e.g., indoor and outdoor concentrations, it is recommended that a strategy be designed in order to obtain a reliable dataset, or at least develop data for estimation of reliable correction factors taking the consequences of the time delay into account. Big variations of ammonia concentration levels between channels may require longer measuring period in each measurement position and consequently the number of reliable dataset for each position will be reduced. Rapid and big variations in the concentrations may require shorter measuring period in each position, thus increasing dependence on correction factors, which will increase the number of data sets and on the other hand increase the uncertainties for each individual data set.

## 4. Conclusions

In the validation of measurement procedures for aerial ammonia levels using an Innova Multi-Gas-Monitor 1312 equipped with an Innova Multipoint Sampler 1303, the following conclusions were drawn:

1. Substantial time delays occur, when the measuring strategy is designed to switch between high and low concentration positions.
2. It is recommended to plan the measuring strategy to allow a minimum of 12.5 to 25 minutes on each position. Even in this case a correction factor may be needed in order to obtain reliable results for further calculations.
3. It is always recommendable to validate the instrument properties with respect to sensibility to variations in concentration levels before starting any data collection.

## Figures and Tables

**Table 1.** Filter setup in the Multi Gas Monitor 1312.

**Gas**	**Filter**	**SIT (s)**	**Centre wavelength (μm)**	**Detection threshold (mg/m^3^)**	**Calibration range (mg/m^3^)**
Carbon Dioxide (CO_2_)	UA0982	5	14.1	1.5	1.5–800 1.5–3,500
Dinitrogen oxide (N_2_O)	UA0985	5	4.5	0.03	0.03–5.45 0.03–50.0
Ammonia (NH_3_)	UA0936	60	9.8	0.2	0.2–74.4
Freon (C_2_H_2_F_4_)	UA0972	5	8.8	0.05	0.05–51.6
Methane (CH_4_)	UA0969	5	8.0	0.4	0.4–45.1 0.4–203.0
